# Diosgenin Prevents Microglial Activation and Protects Dopaminergic Neurons from Lipopolysaccharide-Induced Neural Damage In Vitro and In Vivo

**DOI:** 10.3390/ijms221910361

**Published:** 2021-09-26

**Authors:** Shou-Lun Lee, Ssu-Chieh Tu, Ming-Yen Hsu, Ting-Yu Chin

**Affiliations:** 1 Department of Biological Science and Technology, China Medical University, Taichung 406040, Taiwan; sllee@mail.cmu.edu.tw (S.-L.L.); duhivy@yahoo.com.tw (S.-C.T.); 2 Department of Bioscience Technology, Chung Yuan Christian University, Taoyuan 320314, Taiwan; matt1028ufo@hotmail.com; 3 Department of Chemistry, Chung Yuan Christian University, Taoyuan 320314, Taiwan; 4 Center for Nano Technology, Chung Yuan Christian University, Taoyuan 320314, Taiwan

**Keywords:** diosgenin, neuroprotection, neuroinflammation, lipopolysaccharide, Parkinson’s disease

## Abstract

Background: The prevention of age-related neurodegenerative disorders is an important issue in an aging society. Microglia-mediated neuroinflammation resulting in dopaminergic neuron loss may lead to the pathogenesis of Parkinson’s disease (PD). Lipopolysaccharide (LPS), an endotoxin, induces neuroinflammatory microglial activation, contributing to dopaminergic neuron damage. Diosgenin is a phytosteroid sapogenin with a wide spectrum of pharmacological activities, e.g., anti-inflammatory activity. However, the preventive effect of diosgenin on neuroinflammation is not clear. Thus, in this study, we further investigated the neuroprotective effect of diosgenin on LPS-induced neural damage in vitro and in vivo. Methods: For in vitro experiments, primary mesencephalic neuron-glia cultures and primary microglia cultures isolated from Sprague–Dawley rats were used. Cells were pretreated with diosgenin and then stimulated with LPS. The expression of proinflammatory cytokines or tyrosine hydroxylase (TH) in the cells was analyzed. In vivo, rats were fed a diet containing 0.1% (*w*/*w*) diosgenin for 4 weeks before being administered a unilateral substantia nigra (SN) injection of LPS. Four weeks after the LPS injection, the rats were assessed for lesion severity using the amphetamine-induced rotation test and TH immunohistochemistry. Results: Diosgenin pretreatment prevented LPS-induced neurite shortening in TH-positive neurons in mesencephalic neuron-glia cultures. In addition, pretreatment of primary microglia with diosgenin significantly reduced tumor necrosis factor-α (TNF-α) and inducible nitric oxide synthase (iNOS) expression. Moreover, diosgenin pretreatment significantly suppressed LPS-induced extracellular signal-regulated kinase (ERK) activation. In vivo, the intranigral injection of LPS in rats fed a diosgenin-containing diet significantly improved motor dysfunction and reduced TH expression in SN. Conclusion: These results support the effectiveness of diosgenin in protecting dopaminergic neurons from LPS-induced neuroinflammation.

## 1. Introduction

Neurodegenerative disease derived from population aging is one of the most important public health problems and, as a result, the burden is expected to increase dramatically in both aged and aging societies. Parkinson’s disease (PD) is a human age-related neurodegenerative disease characterized by the neuronal accumulation of α-synuclein cytoplasmic inclusions (commonly referred to as Lewy bodies) and the loss of dopaminergic neurons in the substantia nigra pars compacta. Abnormally deposited α-synuclein activates microglia and, eventually, contributes to a chronic neuroinflammatory state in the central nervous system (CNS) [1]. Neuroinflammation is defined as the organized cellular response to tissue damage and is characterized by activated microglia producing several proinflammatory factors, such as cytokines, proteases and toxic free radicals [2]. Liao et al. indicated that classically activated microglia may release tumor necrosis factor-α (TNF-α), interferon-γ (IFN-γ), interleukin-1β (IL-1β) and IL-6, resulting in a reduction in neurogenesis and dysfunction of the neurotrophic system [3]. The loss of dopamine (DA) is derived from the loss of dopaminergic neurons, which induces a complex series of neurochemical, anatomical and electrophysiological alterations that produce persistent changes in striatal neurons and their signaling pathways [1]. Therefore, microglial activation and neuroinflammation are thought to play important roles in the course of PD.

Microglia are resident immune effector cells in the brain that perform several vital functions in maintaining brain homeostasis [4]. Activated microglia specifically interact with neurons and positively or negatively influence their survival. Uncontrolled overactivation of microglia is a major component of neuroinflammation and has been implicated in various neurodegenerative diseases, such as PD [5]. Thus, inhibition of microglial activation and the subsequent release of proinflammatory mediators are regarded as important therapeutic strategies for neuroinflammation-mediated diseases. Overactivation of microglia is induced by lipopolysaccharide (LPS), IFN-γ, or β-amyloid and results in the excess production of inflammatory cytokines [6]. LPS, a major gram-negative bacterial endotoxin, is a widely described agonist of Toll-like receptor-4 (TLR-4) that stimulates proinflammatory signaling cascades. Moreover, improper regulation of LPS/TLR-4 signaling causes acute sepsis or chronic inflammatory disorders resulting from massive inflammation [7]. According to previous reports, LPS induces the activation of the NF-κB and mitogen-activated protein kinase (MAPK) signaling pathways [8]. MAPKs in mammals include c-Jun NH_2_-terminal kinase (JNK), p38 MAPK and extracellular signal-regulated kinase (ERK), which contribute to the pathology of diverse human diseases [9]. Systemic injection of LPS induces acute systemic inflammation and brain damage [10]. LPS injected into the rat substantia nigra (SN) successfully induces the pathogenesis of PD features in rats, as it elicits an inflammatory response via microglial activation [11]. Furthermore, He et al. indicated that chronic intranasal LPS instillation in mice results in PD [12]. In addition, an epidemiological study recognized airborne particulate matter (PM) as one of the environmental factors potentially involved in PD pathogenesis [13]. Block et al. reported that PM activates microglia, amplifies the microglial response to proinflammatory stimuli and results in cellular damage [14]. Thus, researchers have speculated that neurodegenerative diseases, such as PD, may be strongly associated with ambient air pollution [15].

Diosgenin is an aglycone of the steroidal saponin that is mainly extracted from *Discorea* species and fenugreek seeds. The chemical structure of diosgenin is analogous to those of the sex hormones, such as estrogen, progesterone, testosterone and cortisol, and it is used as the precursor for industrial production of steroidal drugs [16]. Based on accumulating evidence, diosgenin has been pharmacologically evaluated against several cancers, cardiovascular disorders, atherosclerosis, diabetes, allergic diseases, menopausal symptoms and skin aging [17]. In addition, diosgenin has neuroprotective features. Kang et al. indicated that diosgenin reverses functional and structural changes and induces neural regeneration in a diabetic neuropathy model [18]. As shown in the study by Tohda et al., diosgenin treatment not only reduces amyloid plaques and neurofibrillary tangles in the brain [19] but also improves memory and reduces axonal degeneration by activating the membrane-associated rapid response steroid-binding receptor in a mouse Alzheimer’s disease model [20].

PD is the second most common neurodegenerative disease. Neuroprotective strategies may play an important role in preventing the onset and reducing the severity of PD. However, studies have not proven that diosgenin prevents PD. The aim of the present study is to investigate the neuroprotective effects of diosgenin on an LPS-induced PD model. We found that diosgenin pretreatment protected against LPS-induced neural death and microglial activation. Accumulated data from both in vitro and in vivo experiments indicate that diosgenin possesses neuroprotective activity.

## 2. Results

### 2.1. Diosgenin Pretreatment Attenuates LPS-Induced Degeneration of Dopaminergic Neurons in Mesencephalic Neuron-Glia Cultures

**Figure 1:**
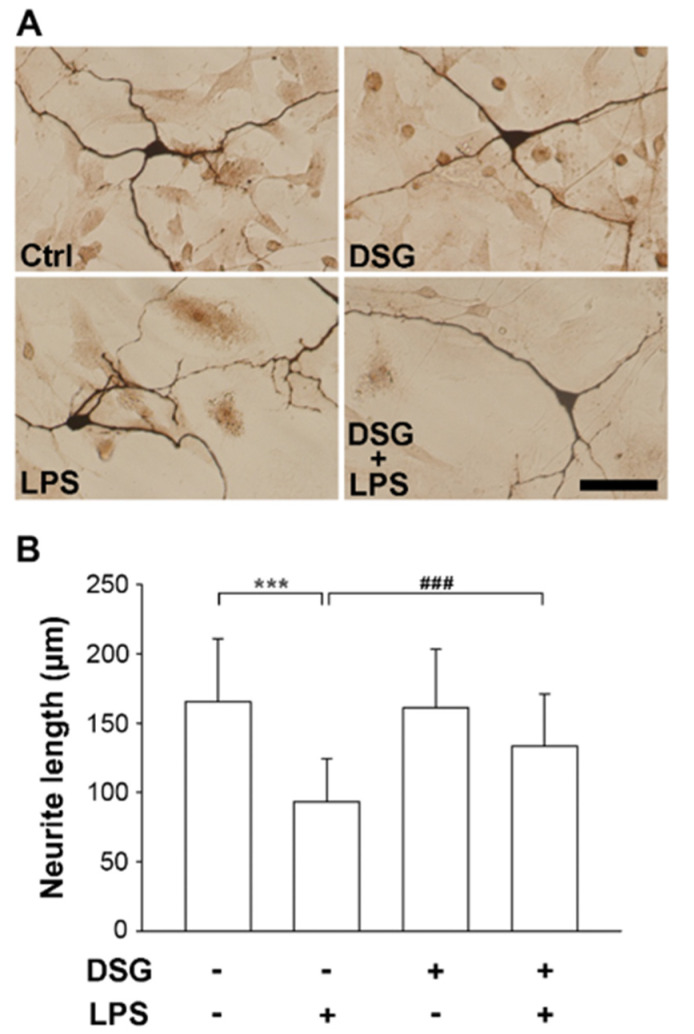
Effects of diosgenin on LPS-induced neurite damage in dopaminergic neurons from mesencephalic neuron-glia cultures. Rat mesencephalic neuron-glia cultures were pretreated with vehicle or diosgenin (2 µM) for 48 h and then treated with or without LPS (25 ng/mL) for 72 h. Representative images of immunostaining for tyrosine hydroxylase (TH) expression in dopaminergic neurons (**A**). Quantification of the neurite length in TH-positive dopaminergic neurons using ImageJ; as many as 50 cells were detected (**B**). The results are presented as the means ± SD. Cultured cells were analyzed in duplicate for each experiment. Three independent experiments (n = 3; duplicate cell preparations from 3 E13.5 embryos of Sprague–Dawley rats). *** *p* < 0.001 compared with the control; ### *p* < 0.001 compared with LPS-treated cultures. The scale bar equals 50 µm.

Degeneration of dopaminergic neurons in the substantia nigra is a hallmark of Parkinson’s disease [21]. We investigated the neuroprotective effects of diosgenin on the degeneration of dopaminergic neurons. Rat midbrain mixed neuron-glia cultures were isolated from embryos (E13.5) and used in this experiment. The cells were pretreated for 48 h with or without diosgenin before treatment with LPS or vehicle. Three days later, the cells were stained using an antibody against tyrosine hydroxylase (TH). TH is the gold standard marker used to identify dopaminergic neurons [22]. Morphologically, the neurites of the TH-immunoreactive neurons in LPS-treated cultures were significantly damaged compared with vehicle-treated control cultures and were characterized by shorter, thinner and broken neurites. Pretreatment with diosgenin significantly resisted LPS-induced neuronal damage (Figure 1A). Furthermore, the neurite length of TH-positive neurons was measured using the ImageJ software. LPS induced a 44% loss of neurite length compared with vehicle-treated cultures. Diosgenin pretreatment prevented the LPS-induced shortening of neurite length, which was significantly increased by 43%, compared with cells cultured in the absence of diosgenin (Figure 1B). These results suggest that LPS-induced damage to dopaminergic neurons is prevented by diosgenin.

### 2.2. Diosgenin Pretreatment Downregulated Inflammation-Associated Genes in LPS-Treated Microglia

**Figure 2:**
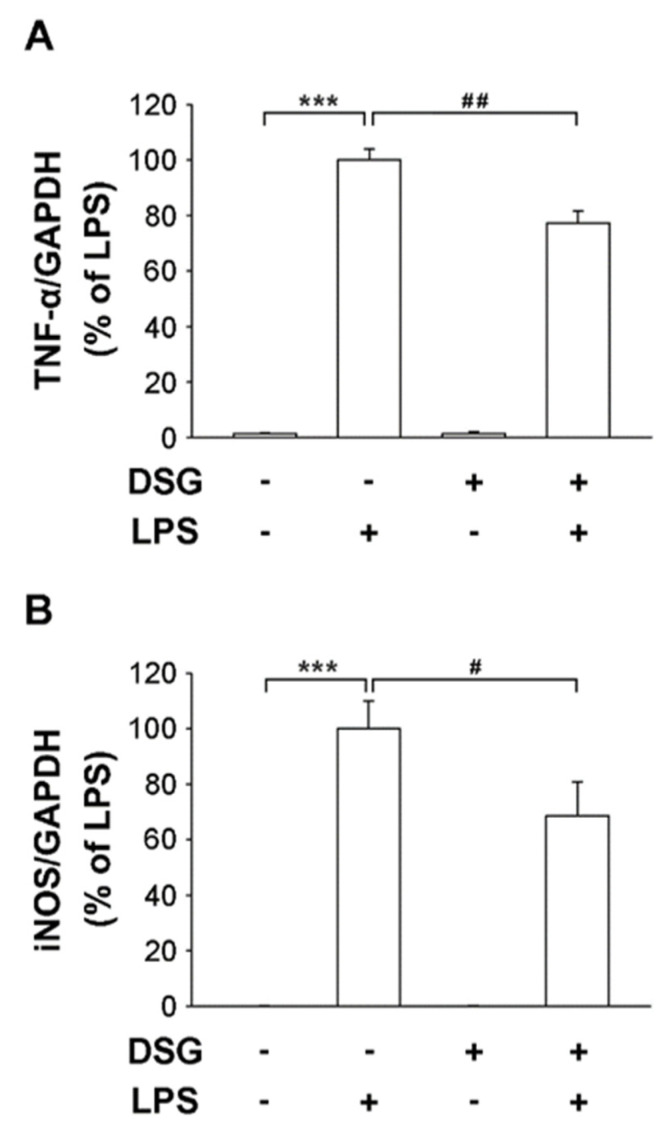
Effects of diosgenin on the LPS-induced release of proinflammatory cytokines from primary rat microglia. Primary rat microglia were treated with vehicle or diosgenin (2 µM) for 30 min before treatment with or without LPS (25 ng/mL) for 4 h. The expression levels of TNF-α (**A**) and iNOS (**B**) measured using real-time RT-PCR were normalized to GAPDH. The results are reported relative to LPS-treated cells as the means ± SD obtained from three independent experiments using different batches of isolated cells. *** *p* < 0.001 compared with the control; # *p* < 0.05 and ## *p* < 0.01 compared with LPS-treated cultures.

Neuroinflammation plays an important role in the progression of Parkinson’s disease. Uncontrolled overactivation of microglia is a major component of neuroinflammation. In this study, we used primary microglial cells isolated from the cerebral cortices of 1-day-old Sprague–Dawley rats. Cells were stimulated with LPS for 4 h and the expression of the proinflammation genes TNF-α and iNOS was significantly increased (Figure 2). However, the expression of the TNF-α and iNOS genes was not affected when the cells were treated with diosgenin alone. Subsequently, the cells were pretreated with diosgenin before treatment with LPS and the expression of TNF-α and iNOS was significantly reduced by 23% and 32%, respectively (Figure 2). Therefore, we speculate that the diosgenin pretreatment suppressed the expression of TNF-α and iNOS in LPS-treated primary rat microglia.

**Figure 3:**
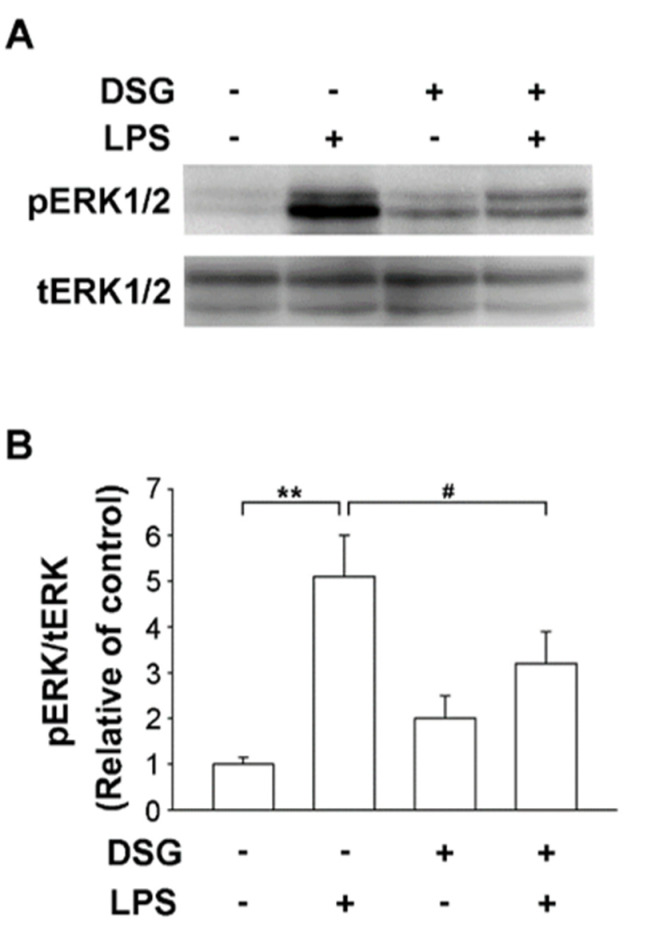
Effects of diosgenin on LPS-induced ERK activation in primary rat microglia. Primary rat microglia were pretreated for 30 min with vehicle or the indicated concentrations of diosgenin before treatment with or without LPS for 30 min. The cells were harvested and phosphorylated ERK 1/2 (pERK) and total ERK 1/2 (tERK) levels were analyzed using Western blotting (**A**). Quantitative densitometry was used to calculate the ratio of pERK to tERK, which was normalized to the control (**B**). The results are presented as the means ± SD obtained from three independent experiments using different batches of isolated cells. ** *p* < 0.01 compared with the control; # *p* < 0.05 compared with LPS-treated cultures.

LPS-induced activation of MAPKs is crucial in the mechanism regulating the production of proinflammatory factors. Numerous studies have confirmed that extracellular signal-regulated kinase (ERK) induces the production of inflammatory mediators [23]. Pretreatment with diosgenin significantly suppressed LPS-induced phosphorylation of ERK1/2 (Figure 3A) and the ratio of pERK to ERK was reduced by 37% (Figure 3B). Thus, the anti-inflammatory effect of diosgenin on LPS-induced microglia suggests an inactive ERK pathway.

### 2.3. Orally Administered Diosgenin Ameliorates Brain Injury Induced by LPS in Rats

**Figure 4:**
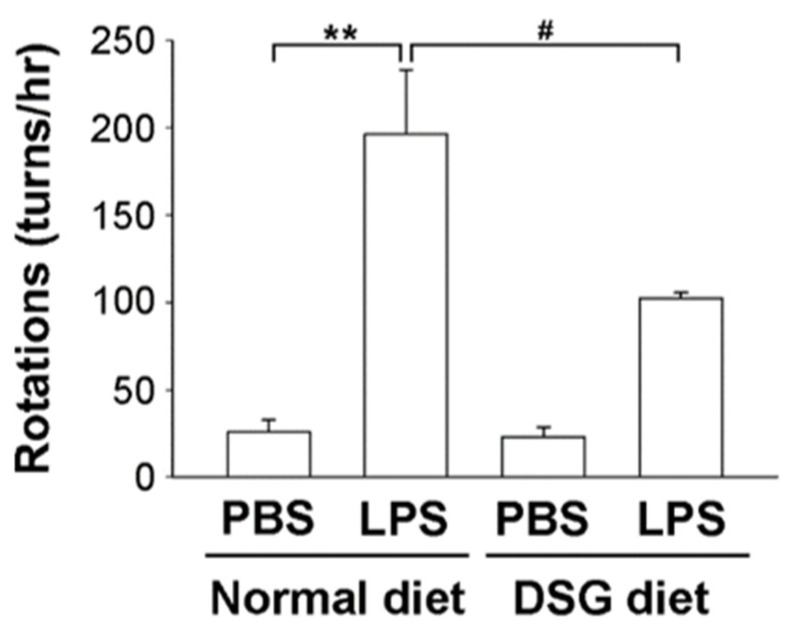
Effects of diosgenin on the amphetamine-induced rotational behavior of a rat model of LPS-induced Parkinson’s disease. Male SD rats were fed diets with or without 0.1% diosgenin (*w*/*w*) for 4 weeks before LPS or PBS was unilaterally injected into the right substantia nigra (SN). After the injection, rats were fed for 4 weeks and then tested for amphetamine-induced rotational behavior. The number of turns induced by amphetamine was recorded for 60 min. The results are presented as the means ± SD obtained from 3 different rats in independent experiments. ** *p* < 0.01 compared with control rats; # *p* < 0.05 compared with LPS-treated rats.

We used a rat model in which LPS was injected unilaterally into the substantia nigra (SN) of the midbrain to investigate whether diosgenin contributes to neuroprotection in vivo. The amphetamine-induced rotational behavioral test showed a significantly increased number of turns by LPS-injected rats compared with PBS-injected rats, regardless of whether the rats were fed a normal diet or a diosgenin diet for 4 weeks (Figure 4). However, diosgenin significantly attenuated the LPS-induced increase in turn numbers by rats (Figure 4). Based on these data, the administration of diosgenin exerts a protective effect on motor dysfunction in rats with LPS-induced brain injury.

**Figure 5:**
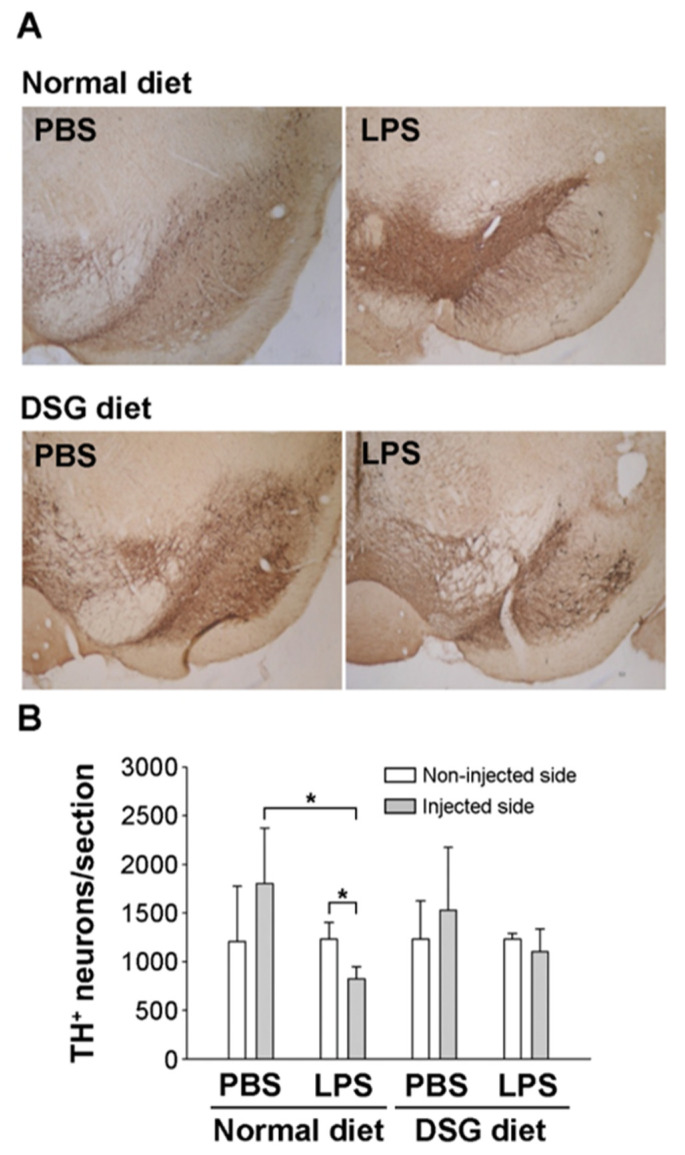
Effects of diosgenin on tyrosine hydroxylase expression in the substantial nigra of a rat model of LPS-induced Parkinson’s disease. Rats with LPS-induced Parkinson’s disease (as shown in Figure 4) were sacrificed after the amphetamine-induced rotation test. SN brain sections were processed for TH immunostaining (**A**). TH-positive neurons in the injected or uninjected SN were calculated using the ImageJ software. (**B**). The results are presented as the means ± SD obtained from 3 different rats in independent experiments. * *p* < 0.05 compared with uninjected SN or compared with PBS-injected SN.

We further investigated the TH expression in the SN by performing an immunohistological analysis after the rotational behavioral assay. In the LPS-injected SN, TH expression was significantly decreased compared with that in the noninjected or PBS-injected SN (Figure 5). In contrast, PBS injection did not alter the TH expression in the SN. However, diosgenin administration dramatically prevented the decrease in TH expression in the SN of animals injected with LPS (Figure 5). Therefore, we speculated that diosgenin might protect dopaminergic neurons from LPS-induced neurotoxicity.

## 3. Discussion

The major finding of this study is that a diosgenin pretreatment ameliorates LPS-induced dopaminergic neuron damage and proinflammatory factors released from microglia. In addition, a diet containing diosgenin may ameliorate motor dysfunction and dopaminergic neuronal injury in rats with a unilateral LPS injection into the SN. Thus, diosgenin protects dopaminergic neurons from LPS-induced damage, possibly by inhibiting microglia-initiated neuroinflammation.

An ounce of prevention is worth a pound of cure. The incidence of neurodegenerative diseases is remarkably increased and associated with an older age. Methods must be developed to prevent or delay neuroinflammation that results in neurodegenerative diseases. LPS, an endotoxin, causes inflammation. Endotoxins in the gut may enter the blood, causing brain inflammation and resulting in neurodegeneration [24]. In addition, PM, an air pollutant, induces neuroinflammation by disrupting the blood–brain barrier, which permits monocyte infiltration, microglial activation and cytokine activity [25]. Neuroinflammation and microglial activation are important factors in the course of PD. Activated microglia accelerate the expression of iNOS and proinflammatory cytokines, which are produced upon LPS binding to TLR4, resulting in the activation of the NF-κB and MAPK signaling pathways [9,26]. Li et al. reported that diosgenin treatment alters the TLR/NF-κB signaling pathway and prevents motor deficits in rats with LPS-induced PD [27]. In addition, diosgenin derivatives exhibit anti-inflammatory activity in LPS-induced microglial BV2 cells by inhibiting of NF-κB and JNK activation [28]. Our results indicate that LPS upregulated the expression of iNOS and TNF-α in primary microglia. Moreover, a microglial pretreatment with diosgenin significantly reduced LPS-induced expression of iNOS and TNF-α and the inactivation of the ERK pathway (Figure 2 and Figure 3). Diosgenin not only prevents neuroinflammation but also protects dopaminergic neurons from LPS-induced damage. According to our data, diosgenin pretreatment avoids dopaminergic neuron damage in mesencephalic neuron-glia cultures treated with LPS (Figure 1). However, a more detailed investigation of the molecular mechanism involved in the anti-neuroinflammatory and neuroprotective effects of diosgenin will be conducted in the future.

Diosgenin has been investigated for the prevention and treatment of various neurological disorders. However, diosgenin is a poorly water-soluble compound that is unstable under physiological conditions and exhibits poor bioavailability and pharmacokinetics, which limit its clinical application. Therefore, diosgenin derivatives have been designed and synthesized to enhance solubility and increase bioavailability for clinical applications [29]. In addition, diosgenin is present in tubers of wild yams. In the study by Tohda et al., healthy adults were orally administered 50 mg of a diosgenin-rich yam extract (corresponding to 8 mg of diosgenin) once daily for 12 weeks and the treatment contributed to an improvement in cognitive function [30]. Herein, we mixed diosgenin into the diets (containing 0.1% diosgenin) and fed rats prior to conducting experiments. Our data showed that pre-feeding diosgenin protected dopaminergic neurons and improved motor dysfunction in rats injected unilaterally with LPS in the SN of the midbrain (Figure 4 and Figure 5). Thus, in vivo data indicated that diosgenin induces neuroprotection against LPS-induced injury. In addition, we plan to investigate whether a mixed diosgenin diet enhances the bioavailability of diosgenin in the future.

In summary, diosgenin pretreatment prevents LPS-induced motor dysfunction and protects dopaminergic neurons by suppressing microglia-mediated neuroinflammation both in vitro and in vivo. Therefore, our data strongly suggest that diosgenin could be developed as a potentially effective pharmacological strategy for preventing or delaying the progression of PD.

## 4. Materials and Methods

### 4.1. Chemicals and Reagents

Antibodies against phosphorylated (p)-ERK and ERK were obtained from Cell Signaling Technology (Beverly, MA, USA). Rabbit anti-tyrosine hydroxylase (TH) antibodies were purchased from Novus Bio. Co. (Littleton, CO, USA). LPS (*Escherichia coli*, serotype 0111:B4) was purchased from Sigma–Aldrich Co. (St. Louis, MO, USA). The 2% Rompun injection was purchased from Bayer (Yongin, Gyeonggi-do, Korea). Zoletil 50 was purchased from VIRBAC (Virbac, Carros, France). All cell culture reagents were purchased from Invitrogen (Carlsbad, CA, USA). All other chemicals were reagent-grade or higher and were purchased from Sigma–Aldrich Co. (St. Louis, MO, USA).

### 4.2. Animal Preparation

Male Sprague–Dawley rats (250–300 g) were purchased from the BioLASCO Taiwan Co., Ltd. (Taipei, Taiwan). Pregnant SD rats were purchased from the National Laboratory Animal Center (Taiwan). The rats were housed in an environmentally controlled animal facility at 22 °C with a daily 12 h light–dark cycle and had free access to regular chow and water ad libitum. The experimental procedures used in the present study were approved by the Institutional Animal Care and Use Committee of Chung Yuan Christian University.

### 4.3. Animal Experimental Design

The unilateral intranigral injection of LPS causes neuroinflammatory response. The neurodegenerative process starts in the substantia nigra pars compacta and consequent dopaminergic denervation of the striatum within 2–4 weeks. This gradual progression of the lesion creates a “4-week concept”. For evaluating the effectiveness of pretreatments aimed to prevent or counteract the progression of nigrostriatal damage, we established a chronic pretreatment with diosgenin for 4 weeks, starting before LPS injection. Feeding with 0.1% (*w*/*w*) diosgenin supplements in chow diet was begun 4 weeks before surgery and continued to the end of experiments.

Two groups of male rats received one of the following diets: normal diet or DSG diet. The normal diet was standard chow (LabDiet 5001; St. Louis, MO, USA) modified to contain 10 g of cellulose (Alphacel nonnutritive bulk) and 12 g of sodium carboxymethyl cellulose per kg of diet. The DSG diet was also a standard chow modified to containing 1 g of diosgenin, 9 g of cellulose and 12 g of carboxymethyl cellulose per kg of diet. Rats were orally administered chow mixed with or without 0.1% (*w*/*w*) diosgenin for 4 weeks. Then, each group was randomly divided into two subgroups that received an injection of PBS or LPS. LPS (or PBS) was injected unilaterally into the SN as previously described [31]. Briefly, the rats were placed in a stereotaxic apparatus and administered a unilateral injection of LPS (5 μg in 3 μL PBS) or PBS into the right SN (anteroposterior (AP), 5.3 mm, mediolateral (ML), 2.3 mm and dorsoventral (DV), 7.7 mm from the bregma), after anesthesia was achieved with Zoletil 50 (50 mg/kg) and 2% Rompun injection (0.1 mL/kg). Infusions of LPS or PBS were performed at a rate of 1 μL/min for LPS or PBS. Then, the needle was left in place for 5 min to avoid reflux along the injection track. Four weeks after the LPS injection, all animals underwent behavioral evaluation.

### 4.4. Mesencephalic Neuron-Glia Cultures

Mesencephalic tissues were dissected from 13.5-day-old embryos and dissociated by gentle mechanical trituration. Cells were suspended in maintenance medium consisting of MEM supplemented with 10% FBS, 10% horse serum, 1 g/L glucose, 2 mM L-glutamine, 1 mM sodium pyruvate, 100 μM nonessential amino acids, 100 U/mL penicillin and 100 μg/mL streptomycin in plates (10^5^ cells/mL) at 37 °C in a humidified atmosphere composed of 5% CO_2_ and 95% air. Fresh medium was added to each well 3 days later. Six-day-old cultures were used for treatment.

### 4.5. Primary Culture of Microglia

Microglia were isolated and purified from whole brains of neonatal (1-day-old) Sprague–Dawley rats. Briefly, the brains were dissected from the embryos, cut into small pieces and stably treated with the digestion solution containing 30 mg/mL papain, 50 mM EDTA, 2 mg/mL cysteine and 150 mM CaCl_2_. The dissected cells were reacted with DNase I, added to 10% horse serum, collected by centrifugation and resuspended in a serum-free medium containing DMEM-F12 containing 10% FBS, 2 mM L-glutamine, 1 mM sodium pyruvate, 100 μM nonessential amino acids, 100 U/mL penicillin and 100 μg/mL streptomycin in plates (10^5^ cells/mL) at 37 °C in a humidified atmosphere of 5% CO_2_ and 95% air. The medium was changed 3 days later. Two weeks later, microglia were separated from astrocytes by shaking the plates at 200 rpm for 4 h.

### 4.6. Real-Time Quantitative Reverse-Transcription Polymerase Chain Reaction (qRT-PCR)

The cells were detached and thoroughly washed with cold PBS. RNA was extracted using TRIzol Reagent according to the manufacturer’s protocol (Invitrogen, Carlsbad, CA, USA). Total RNA (1 μg) was reverse-transcribed into cDNA with SuperScript III Reverse Transcriptase (Invitrogen) using random primers. qRT-PCR was performed using FastStart Universal SYBR Green Master Mix (Rox) (Roche AG, Mannheim, Germany). Thermocycling was performed with a 7300 Real Time 40 PCR system (Applied Biosystems, Foster City, CA, USA). Conditions for qRT-PCR included initial denaturation at 94 °C for 1 min, followed by 98 °C for 15 s and 60 °C for 40 cycles. The primers used in this study were as follows: TNFα, forward 5′-CTCACACTCAGATCATCTTCT-3′ and reverse 5′-GGTATGAAATGGCAAATCGG-3′; iNOS, forward 5′-GGTATGAAATGGCAAATCGG-3′ and reverse 5′-CGGACCATCTCCTGCATT-3′; GAPDH, forward 5′-GCAAGAGAGAGGCCCTCA-3′ and reverse 5′-TGTGAGGGAGATGCTCAGTG-5′. The relative expression of each gene was determined and compared to the expression of GADPH to eliminate the effect of the cell population.

### 4.7. Western Blot Analysis

Microglial cells were preincubated with 2 μM diosgenin for 30 min before 25 ng/mL LPS was added to the cells. Levels of the p-ERK1/2 and ERK1/2 proteins were detected in whole cell lysates 30 min after LPS treatment. Equal amounts of protein (30 μg) were separated on 10% SDS–PAGE gels, transferred to PVDF membranes using an electrophoretic transfer system and subjected to immunoblotting with the following specific primary antibodies for 1 h at room temperature (RT): rabbit-p-ERK1/2 and rabbit-ERK1/2 (1:1000). After washing, the membranes were incubated with secondary antibodies (1:4000; Jackson ImmunoResearch Laboratories, West Grove, PA, USA) for 1 h at RT. Finally, the blots were developed with enhanced chemiluminescence detection reagents (Amersham Biosciences, Amersham, Buckingham Shire, UK).

### 4.8. Rotational Behavioral Assay

Four weeks after the LPS injection, rats were administered amphetamine (2 mg/kg, i.p.) in order to assess lesion severity. For this experiment, the rats were placed in individual plastic hemispherical bowls and injected with amphetamine following a short period of acclimation. Turns were enumerated at 30 min post-injection and recorded over a 60 min observation period.

### 4.9. Tyrosine Hydroxylase Immunohistological Staining

After the behavioral tests, rats were deeply anesthetized and transcardially perfused with 4% paraformaldehyde. The rat brains were stored in a 30% sucrose solution at 4 °C until they sank, at which time samples were frozen and sectioned on a sliding microtome. Frozen tissues were cut into 30 μm thick sections and processed for immunohistochemistry. SN sections or cells were then rinsed with TBS and subsequently incubated with 0.2% hydrogen peroxide (in TBS) for 10 min. Following TBS rinses, samples were incubated with the blocking solution (TBS containing 2% bovine serum albumin and 0.1% Triton X-100) for 1 h. Next, samples were incubated with a primary antibody (1:500; goat anti-rabbit) for 1 h. Samples were washed again with TBST, reacted with ABC complex at RT for 1 h, washed with TBST and finally developed with 3,3-diaminobenzidine (DAB). Stained samples were analyzed under a bright-field microscope.

### 4.10. Statistical Analyses

Data are presented as the means ± standard deviation (SD) of three independent experiments. The significance of differences in the mean values was assessed using the unpaired Student’s *t*-test. Statistical significance was defined as *p* < 0.05 compared with the appropriate control group. The statistical software used for analyses was SigmaPlot (version 11, San Jose, CA, USA).

## Data Availability

The data presented in this study are available on request from the corresponding author.

## Abbreviations

CNS	central nervous system
DA	dopamine
DSG	DSG
ERK	extracellular signal-regulated kinase
IFN-γ	interferon-γ
IL-1β	interleukin-1β
iNOS	inducible nitric oxide synthase
JNK	c-Jun NH2-terminal kinase
LPS	lipopolysaccharide
MAPKs	mitogen-activated protein kinases
PD	Parkinson’s disease
PM	particulate matter
SN	substantia nigra
TH	tyrosine hydroxylase
TLR-4	Toll-like receptor-4
TNF-α	tumor necrosis factor-α

## References

[1] Nayak D., Roth T.L., McGavern D.B. (2014). Microglia development and function. Annu. Rev. Immunol..

[2] Yuste J.E., Tarragon E., Campuzano C.M., Ros-Bernal F. (2015). Implications of glial nitric oxide in neurodegenerative diseases. Front. Cell Neurosci..

[3] Liao B., Zhao W., Beers D.R., Henkel J.S., Appel S.H. (2012). Transformation from a neuroprotective to a neurotoxic microglial phenotype in a mouse model of ALS. Exp. Neurol..

[4] Matt S.M., Johnson R.W. (2016). Neuro-immune dysfunction during brain aging: New insights in microglial cell regulation. Curr. Opin. Pharmacol..

[5] Graeber M.B., Streit W.J. (1990). Microglia: Immune network in the CNS. Brain Pathol..

[6] Kettenmann H., Kirchhoff F., Verkhratsky A. (2013). Microglia: New roles for the synaptic stripper. Neuron.

[7] Lu Y.C., Yeh W.C., Ohashi P.S. (2008). LPS/TLR4 signal transduction pathway. Cytokine.

[8] Gao M., Chen L., Yu H., Sun Q., Kou J., Yu B. (2013). Diosgenin down-regulates NF-κB p65/p50 and p38MAPK pathways and attenuates acute lung injury induced by lipopolysaccharide in mice. Int. Immunopharmacol..

[9] Kim E.K., Choi E.J. (2015). Compromised MAPK signaling in human diseases: An update. Arch. Toxicol..

[10] Jeong H.K., Jou I., Joe E.H. (2010). Systemic LPS administration induces brain inflammation but not dopaminergic neuronal death in the substantia nigra. Exp. Mol. Med..

[11] Herrera A.J., Castano A., Venero J.L., Cano J., Machado A. (2000). The single intranigral injection of LPS as a new model for studying the selective effects of inflammatory reactions on dopaminergic system. Neurobiol. Dis..

[12] He Q., Yu W., Wu J., Chen C., Lou Z., Zhang Q., Zhao J., Wang J., Xiao B. (2013). Intranasal LPS-mediated Parkinson’s model challenges the pathogenesis of nasal cavity and environmental toxins. PLoS ONE.

[13] Heusinkveld H.J., Wahle T., Campbell A., Westerink R.H.S., Tran L., Johnston H., Stone V., Cassee F.R., Schins R.P.F. (2016). Neurodegenerative and neurological disorders by small inhaled particles. Neurotoxicology.

[14] Block M.L., Elder A., Auten R.L., Bilbo S.D., Chen H., Chen J.C., Cory-Slechta D.A., Costa D., Diaz-Sanchez D., Dorman D.C. (2012). The outdoor air pollution and brain health workshop. Neurotoxicology.

[15] Genc S., Zadeoglulari Z., Fuss S.H., Genc K. (2012). The adverse effects of air pollution on the nervous system. J. Toxicol..

[16] Djerassi C. (1992). Drugs from Third World plants: The future. Science.

[17] Chen Y., Tang Y.M., Yu S.L., Han Y.W., Kou J.P., Liu B.L., Yu B.Y. (2015). Advances in the pharmacological activities and mechanisms of diosgenin. Chin. J. Nat. Med..

[18] Kang T.H., Moon E., Hong B.N., Choi S.Z., Son M., Park J.H., Kim S.Y. (2011). Diosgenin from *Dioscorea nipponica* ameliorates diabetic neuropathy by inducing nerve growth factor. Biol. Pharm. Bull..

[19] Tohda C., Urano T., Umezaki M., Nemere I., Kuboyama T. (2012). Diosgenin is an exogenous activator of 1,25D_3_-MARRS/Pdia3/ERp57 and improves Alzheimer’s disease pathologies in 5XFAD mice. Sci. Rep..

[20] Tohda C., Lee Y.A., Goto Y., Nemere I. (2013). Diosgenin-induced cognitive enhancement in normal mice is mediated by 1,25D_3_-MARRS. Sci. Rep..

[21] Olanow C.W., Tatton W.G. (1999). Etiology and pathogenesis of Parkinson’s disease. Annu. Rev. Neurosci..

[22] White R.B., Thomas M.G. (2012). Moving beyond tyrosine hydroxylase to define dopaminergic neurons for use in cell replacement therapies for Parkinson’s disease. CNS Neurol. Disord. Drug Targets.

[23] Yuan L., Liu S., Bai X., Gao Y., Liu G., Wang X., Liu D., Li T., Hao A., Wang Z. (2016). Oxytocin inhibits lipopolysaccharide-induced inflammation in microglial cells and attenuates microglial activation in lipopolysaccharide-treated mice. J. Neuroinflamm..

[24] Brown G.C. (2019). The endotoxin hypothesis of neurodegeneration. J. Neuroinflamm..

[25] Arias-Pérez R.D., Taborda N.A., Gomez D.M., Narvaez J.F., Porras J., Hernandez J.C. (2020). Inflammatory effects of particulate matter air pollution. Environ. Sci. Pollut. Res. Int..

[26] Glass C.K., Saijo K., Winner B., Marchetto M.C., Gage F.H. (2010). Mechanisms underlying inflammation in neurodegeneration. Cell.

[27] Li B., Xu P., Wu S., Jiang Z., Huang Z., Li Q., Chen D. (2018). Diosgenin Attenuates Lipopolysaccharide-Induced Parkinson’s Disease by Inhibiting the TLR/NF-κB Pathway. J. Alzheimers Dis..

[28] Cai B., Seong K.J., Bae S.W., Chun C., Kim W.J., Jung J.Y. (2018). A synthetic diosgenin primary amine derivative attenuates LPS-stimulated inflammation via inhibition of NF-κB and JNK MAPK signaling in microglial BV2 cells. Int. Immunopharmacol..

[29] Cai B., Zhang Y., Wang Z., Xu D., Jia Y., Guan Y., Liao A., Liu G., Chun C., Li J. (2020). Therapeutic Potential of Diosgenin and Its Major Derivatives against Neurological Diseases: Recent Advances. Oxid. Med. Cell. Longev..

[30] Tohda C., Yang X., Matsui M., Inada Y., Kadomoto E., Nakada S., Watari H., Shibahara N. (2017). Diosgenin-Rich Yam Extract Enhances Cognitive Function: A Placebo-Controlled, Randomized, Double-Blind, Crossover Study of Healthy Adults. Nutrients.

[31] Cheng C.Y., Barro L., Tsai S.T., Feng T.W., Wu X.Y., Chao C.W., Yu R.S., Chin T.Y., Hsieh M.F. (2021). Epigallocatechin-3-Gallate-Loaded Liposomes Favor Anti-inflammation of Microglia Cells and Promote Neuroprotection. Int. J. Mol. Sci..

